# Social determinants of health and adolescent childbearing in WHO Eastern Mediterranean countries

**DOI:** 10.1186/s12939-023-01861-2

**Published:** 2023-05-02

**Authors:** Abdesslam Boutayeb

**Affiliations:** Laboratory of Stochastic and Deterministic Modelling, Department of Mathematics, Faculty of Sciences, University Mohamed Premier, Boulevard Mohamed VI, Oujda, 60 000 Morocco

**Keywords:** Adolescent pregnancy, Motherhood, Social determinants, Inequity, Milieu, Education, Wealth.

## Abstract

**Objectives:**

Teenage pregnancy and motherhood is a crucial problem in countries of the World Health Organisation Eastern Mediterranean Region (WHO–EMR). The aim of this paper is to describe and analyse the phenomenon of adolescent childbearing in ten countries according to social determinants like milieu (rural–urban), education level, wealth quintiles, territoriality (countries, regions) and nationality.

**Methods:**

Inequity in terms of adolescent childbearing was analysed using disaggregated data given by Demographic Health Surveys (DHS), UNICEF Multiple Indicator Cluster Surveys (MICS) and the Pan Arab Project for Family Health (PAPFAM) surveys. Beside the absolute differences (gaps) and relative differences (ratios), the index of dissimilarity (ID) was used to compare the distributions of adolescent pregnancy and motherhood by social determinants in each country.

**Results:**

Data analysis indicates that the average percentage of adolescent women aged 15–19 years who have begun childbearing shows a large difference between countries, varying from 0.4% in Tunisia to 15.1% in Sudan, combined with huge gaps within each country as indicated by the values of the index of dissimilarity. Poor, rural and non-educated adolescent girls are more exposed to teenage childbearing than their counterparts—rich, urban and educated girls.

**Conclusion:**

According to different social determinants, sensible variations are seen in terms of adolescent pregnancy and motherhood within the ten countries considered in this study. This is a clear appeal to decision makers to reduce child marriage and pregnancy by acting on social determinants of health, targeting disadvantaged girls coming mainly from marginalised groups and poor families living in remote rural zones.

## Introduction

According to the World Health Organisation (WHO), among adolescent girls aged 15–19 years, some 21 million become pregnant and around 12 million of them give birth while more than 10 million unintended pregnancies and nearly four million unsafe abortions are registered each year in developing countries [1]. Sub-Saharan African countries lead the world in adolescent pregnancies [2]. According to statistics released by the World Bank in 2019, the top ten countries with the highest adolescent fertility rate (births per 1,000 women ages 15–19), were Niger (180), Mali (165), Chad (155), Equatorial Guinea (151), Angola (145), Mozambique (144), Malawi (132), Guinea (131), Central African Republic (125) and Congo Democratic Republic (121) [3].

In 2018, a systematic review on *“Determinants of adolescent pregnancy in sub-Saharan Africa*” was published by Yakubu and Salisu. The authors selected and analysed 24 studies from nine sub-Saharan African countries (Ethiopia, Ghana, Kenya, Nigeria, South Africa, Swaziland, Tanzania, Zambia and Uganda). They concluded that adolescent pregnancies were influenced mainly by three kinds of factors, namely: **(1)** Socio-cultural, environmental and economic factors: (poverty, religion, early marriage, coercive sexual relations, …), **(2)** Individual factors (excessive use of alcohol, substance abuse, educational status, …) and **(3)** Health service-related factors (cost of contraceptives, inadequate and unskilled health workers, long waiting time and lack of privacy at clinics, misconceptions about contraceptives, …) [4].

Noting that adolescent fertility rates in Latin America and the Caribbean (LAC) remain unacceptably high and that, in 2013, LAC was the only region with a rising trend in pregnancies in adolescents younger than 15 years, a technical consultation with global, regional and country-level stakeholders was held by PAHO/WHO, UNFPA and UNICEF in order to take stock of the situation and agree on strategic approaches and priority actions to accelerate progress on adolescent pregnancy. The paper published by Caffe et al. in 2017 included the main conclusions of the group and especially the seven priority action areas, namely: **(1)** Make adolescent pregnancy, its drivers and impact, and the most affected groups more visible with disaggregated data and stories, **(2)** Design interventions targeting the most vulnerable groups, ensuring the approaches are adapted to their local realities and address their specific challenges, **(3)** Engage and empower youth to contribute to the design, implementation and monitoring of strategic interventions, **(4)** Abandon ineffective interventions and invest in applying proven ones, **(5)** Strengthen inter-sectoral collaboration to effectively address the drivers of adolescent pregnancy in LAC, **(6)** Move from boutique projects to large-scale and sustainable programmes, and **(7)** Create an enabling environment for gender equality and adolescent sexual and reproductive health and rights [5].

**Fig. 1:**
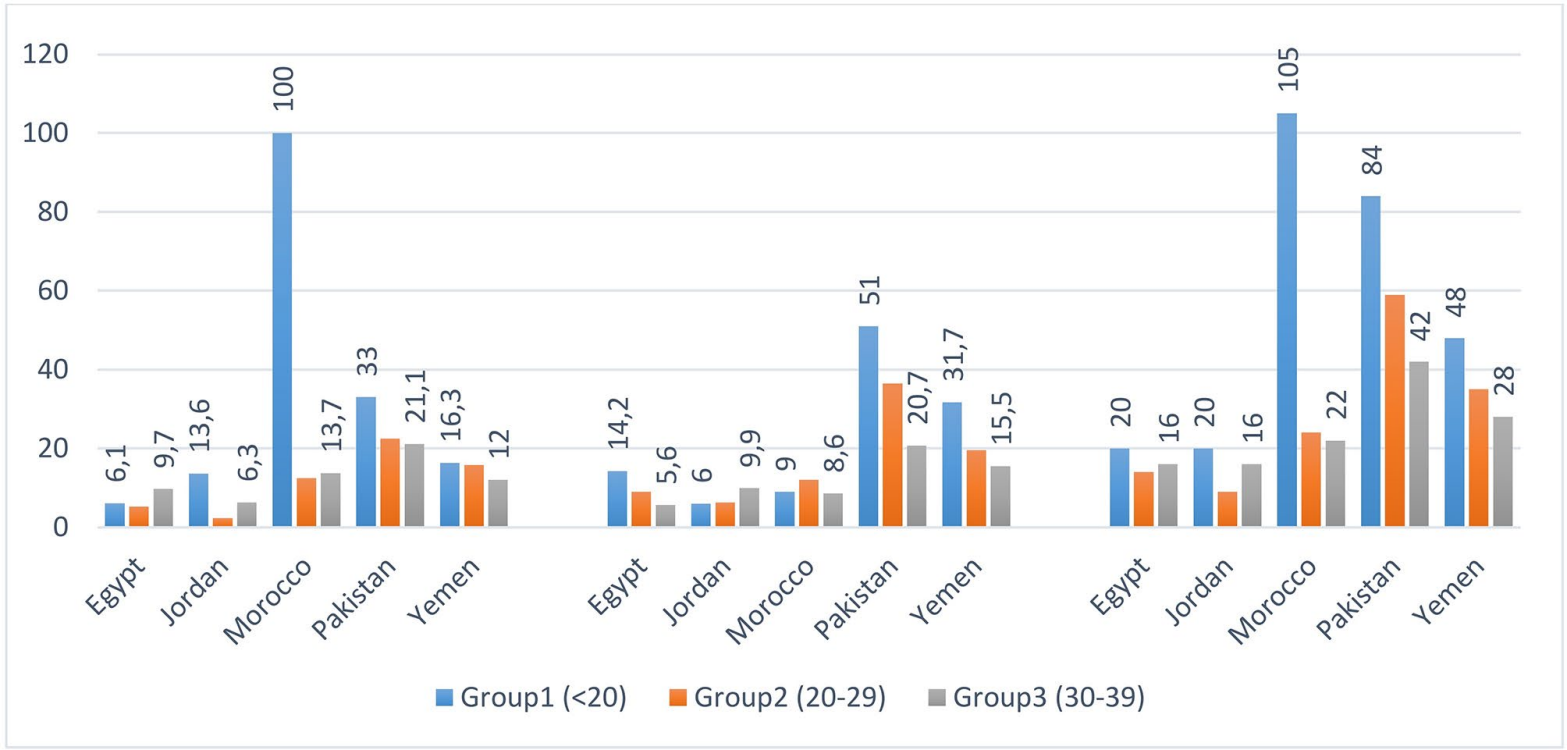
Perinatal mortality by mother’s age at birth in five countries of the WHO–EMR. **Data source**: DHS (Egypt 2014, Jordan 2017–18, Pakistan 2017–18, Yemen 2013) and PAPFAM Morocco 2018.

The phenomenon of early marriage and adolescent childbearing is a crucial social and medical problem challenging health decision makers in countries of the WHO Eastern Mediterranean Region (WHO–EMR). For instance, the numbers of stillbirths and early neonatal deaths as well as the perinatal mortality rate are generally much higher in Group 1 of young married women aged less than 20 years than among married women of Group 2 (aged 20–29) or Group 3 (30–39 years) [6–10]. Indeed, Fig. 1 shows that the ratio between perinatal mortality rate in Group 1 and perinatal mortality rate in Group 2 or Group 3 is 1.4, 2.2, 4.7, 2 and 1.7 in Egypt, Jordan, Morocco, Pakistan and Yemen, respectively. For the number of stillbirths, the ratio reaches 8 in Morocco and nearly 6 in Jordan. In Egypt, Pakistan and Yemen, the number of early neonatal deaths in Group 1 are 2.5, 2.4 and 2 times higher than in Group 2 or Group 3 while nearly the same number of early neonatal deaths is seen between Group 1 (6) and Group 3 (6.3) in Jordan and between Group 1 (9) and Group 3 (8.6) in Morocco.

**Fig. 2:**
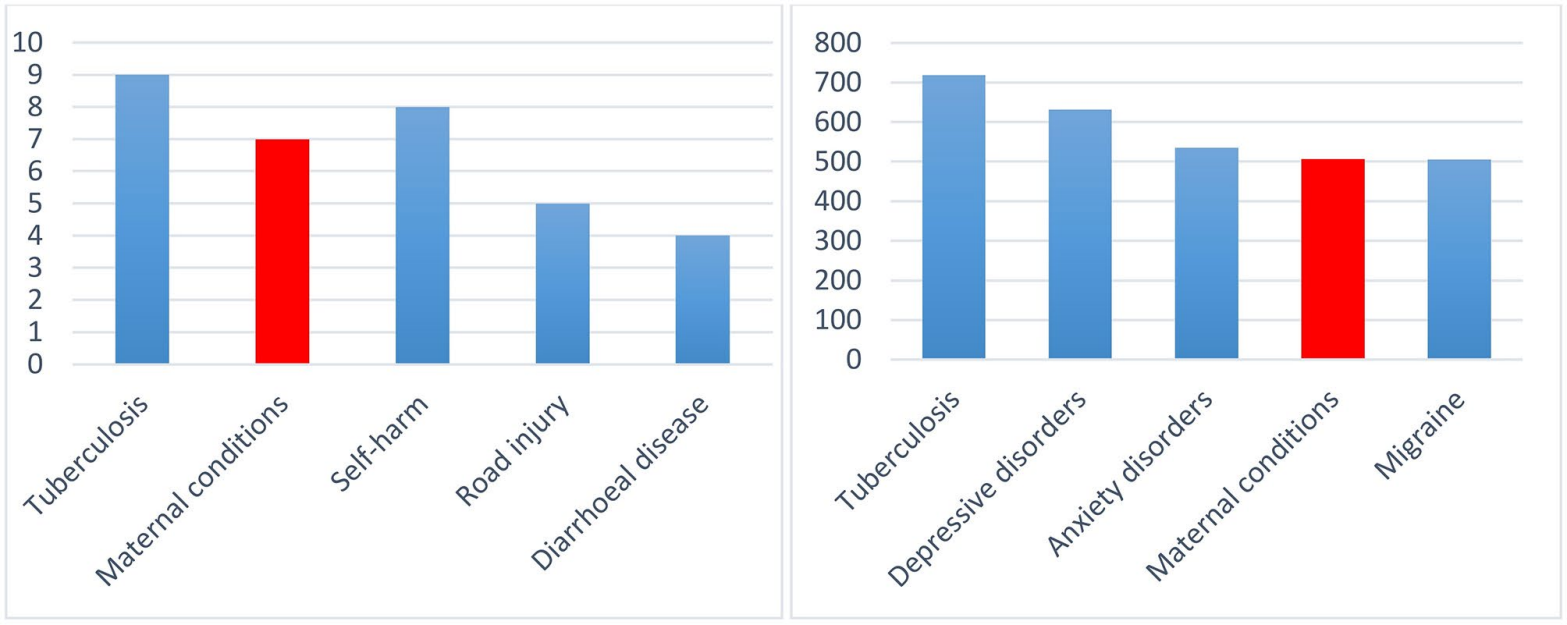
Top 5 causes of mortality and DALYs among girls aged 15–19 in the world **Source**: UNICEF [11].

*According to UNICEF*, *“Adolescent girls, especially those in early adolescence, are particularly vulnerable to the health consequences of pregnancy and delivery as their bodies may not be physically ready. Obstetric fistula, eclampsia, puerperal endometritis and systemic infections are just some of the serious conditions that they may face in the short- and long-term”.* Figure 2 shows that, globally, maternal conditions are among the top causes of mortality and disability-adjusted life years (DALYs) among girls aged 15–19) [11].

Moreover, beyond affecting seriously health indicators like maternal and infant mortality, early marriage is a violation of human rights affecting seriously the life of adolescent girls at least at three levels: **(1)** early married girls are highly exposed to all kinds of violence (physical, psychological, sexual), school dropout, exclusion from training programmes and violation of individual freedom, **(2)** by propelling girls into motherhood before they are physically, emotionally or financially ready, adolescent pregnancy profoundly affects girls’ life trajectories, limiting their educational attainment and their earning potential, thereby increasing the likelihood of poverty and perpetuating intergenerational cycles of poverty [5, 11, 12], and **(3)** adolescent girls who are already marginalized are often disproportionately affected by early pregnancy, due to its interdependencies with poverty, social exclusion, sexual violence and child marriage, and their limited access to comprehensive sexuality education and sexual and reproductive health services including contraceptive information, counselling and services [5, 12, 13].

As stressed by the WHO Commission on Social Determinants of Health (CSDH) in its report released in 2008 under the title *“Closing the gap in a generation: health equity through action on the social determinants of health”*, social determinants of health are defined as the conditions in which people are born, grow, live, work and age. Social determinants of health are seen to be responsible for unfair and avoidable health inequities within and between countries [14]. In 2021, the Commission on Determinants of Health in the Eastern Mediterranean Region (CSDH–EMR) published its report *“Build back fairer: achieving health equity in the Eastern Mediterranean Region”* showing the urgent necessity for WHO Eastern Mediterranean countries to reduce health inequities by acting on social determinants of health. Taking into account the fact that the WHO–EMR includes countries with the highest income per capita in the world such as Kuwait, Qatar and the UAE and countries among the poorest in the world such as Afghanistan, Djibouti and Yemen, the Commission suggested that, although at different levels, all countries of the WHO–EMR should reduce health inequities following the formula: do something, do more, do better [15]. Reducing inequalities in early marriage and adolescent pregnancy/motherhood by acting on social determinants of health was, however, given very little consideration in the report of the WHO Eastern Mediterranean Region Commission.

The percentage of young women who have begun childbearing (those who have had a live birth or were pregnant at the period of the survey) shows huge gaps between and within countries of this region according to social determinants of health (SDH) like milieu of residence (urban–rural), level of education, wealth quintiles, territoriality (regions) and nationality/ethnicity. Consequently, it is interesting to analyse the effect of social determinants on early marriage and adolescent pregnancy in the 22 countries of the WHO Eastern Mediterranean region (Afghanistan, Bahrain, Djibouti, Egypt, Iran, Iraq, Jordan, Kuwait, Lebanon, Libya, Morocco, Oman, Pakistan, Palestine, Qatar, Saudi Arabia, Somalia, Sudan, Syria, Tunisia, United Arab Emirates (UAE) and Yemen). Unfortunately, this task can only be achieved partially due to unavailability and scarcity of disaggregated data in most countries of the WHO–EMR.

In 2011, the World Health Organisation published the *WHO Guidelines on preventing early pregnancy and poor reproductive outcomes among adolescents in developing countries.* The main objective of this publication was to provide recommendations on action and research for (a) preventing early pregnancy: by preventing marriage before 18 years of age; by increasing knowledge and understanding of the importance of pregnancy prevention; by increasing the use of contraception; and by preventing coerced sex; (b) preventing poor reproductive outcomes: by reducing unsafe abortions; and increasing the use of skilled antenatal, childbirth and postnatal care [16]. Four strong recommendations were suggested, namely: **(1)** Encourage political leaders, planners and community leaders to formulate and enforce laws and policies to prohibit marriage of girls before 18 years of age, **(2)** Undertake interventions to delay marriage of girls until 18 years of age by influencing family and community norms. These interventions should be undertaken in conjunction with interventions directed at political leaders/planners, **(3)** Implement interventions to inform and empower girls, in combination with interventions to influence family and community norms, to delay the age of marriage among girls under 18 years of age, and **(4)** Increase educational opportunities for girls through formal and non-formal channels, to delay marriage until 18 years of age. These strong recommendations were accompanied by recommendations for further research [16].

Building on the different recommendations reported from various sources including the World Health Organisation, UNICEF, UNFPA, PAHO and researchers dealing with adolescent marriage and childbearing in Africa and Latin America and the Caribbean, we devote this paper to the description and analysis of the effect of different social determinants of health on the crucial problem of adolescent pregnancy and motherhood in ten selected countries of the WHO–EMR where disaggregated data by milieu of residence (rural–urban), level of education, wealth quintiles, territoriality (regions) and nationality are available. Our aim is to show how scientific research can be applied to identify pathways leading to the issue of adolescent childbearing and hence propose pragmatic and efficient strategies susceptible to end or at least to limit the burden of this crucial problem. Adolescent childbearing is a multidimensional problem and its reduction needs a regular collaborative action involving international organisations (United Nations, WHO, UNICEF, UNFPA, Human Rights organisations…), national sectors (justice, education, health, research, employment…), local actors (municipalities, communes, ONGs).

To our knowledge, there is no published paper dealing with adolescent childbearing, social determinants of health and inequity in the way proposed in the present work.

## Materials and methods

Inequity in terms of adolescent pregnancy and motherhood is analysed using disaggregated (secondary) data as given by Demographic Health Surveys (DHS), the UNICEF Multiple Indicator Cluster Surveys (MICS) and the Pan Arab Project for Family Health (PAPFAM) surveys. Sampling techniques are those usually used in household surveys by Demographic Health Surveys. For each survey, the number of households surveyed and the number of women surveyed individually in urban and rural areas are given in Table 1.

**Table 1:** Number of households and women surveyed in each survey

Population Country	Households surveyed Urban Rural Total	women surveyed Urban Rural Total	% of women 15–19 who had begun childbearing
Egypt DHS 2014	13,962 14,213 28,175	9628 12,134 21,762	10.9%
Iraq MICS 2018	13,876 6338 20,214	20,449 10,211 30,660	13.2%
Jordan DHS 2017–2018	14,944 3858 18,802	11,745 2944 14,689	5.2%
Morocco PAPFAM 2018	8788 6234 15,022	5528 4441 9969	5.1%
Pakistan DHS 2017–2018	6091 5778 11,869	6098 6266 12,364	8.1%
Sudan MICS 2014	4825 11,976 16,801	5979 12,323 18,302	15.1%
Tunisia MICS 2018	7662 3563 11,225	7004 3555 10,559	0.4%
Yemen DHS 2013	4693 12,658 17,351	4548 12,108 16,656	10.7%
Palestine MICS 2019–2020	5516 2138 9326 Camps: 1672	6584 2484 11,135 Camps: 2067	5.8%
Qatar MICS 2014	Qatari Non-Qatari Total 2235 2266 4501	Qatari Non-Qatari Total 3419 2280 5699	1.4%

The index of dissimilarity (ID) is used to compare the distribution of adolescent childbearing by milieu of residence, education level, wealth quintile, regions and nationality in each country. This index is a commonly-used method of measuring inequality between two populations according to geographic, demographic and socio-economic factors. It compares how evenly one population sub-group A is spread out compared to another population sub-group B. The ID is calculated as follows:

$${\rm ID } = \frac{1}{2}\sum _{\text{i}=1}^{\text{N}}\left|\frac{\text{a}\text{i}}{\text{A}}- \frac{\text{b}\text{i}}{\text{B}}\right|,$$

where A is the sub-group A population and a.i. represents the sub-group A population in category i. Similarly, B is the sub-group B population and bi is the sub-group A population in category i,

The index of dissimilarity has a value between 0 and 1 (also given as a percentage between 0% and 100%). A value of ID near zero means that the distribution between the two groups shows no (or little) difference while a value of ID approaching 1 indicates an inequitable distribution between the two groups.

## Results

Data provided by Demographic Health Surveys in Egypt (2014) [6], Jordan (2017–18) [7], Pakistan (2017–18) [8] and Yemen (2013) [9]; data provided by the Pan Arab Project for Family Health surveys in Morocco (2018, 2011) [10, 17]; and finally data provided by Mixed Indicator Cluster Surveys in Iraq (2018) [18], Palestine (2014) [19], Qatar (2012) [20], Sudan (2014) [21] and Tunisia (2018) [22] indicate that the average percentage of adolescent women aged 15–19 years who have begun childbearing varies considerably between countries. The distribution follows a steepest gradient (0.4%, 2.2%, 5.1%, 5.2%, 6.9%, 8.1%, 10.7%, 10.9%, 13.2% and 15.1%), showing that adolescent girls in Sudan (15.1%) are nearly 38 times more exposed to pregnancy and motherhood than their counterparts in Tunisia (0.4%).

Similarly, huge gaps are seen within each country according to different social determinants such as milieu of residence (urban–rural), level of education, wealth quintiles, territoriality (regions) and nationality.

### Adolescent childbearing by milieu (rural–urban) in 9 countries of the WHO–EMR

**Fig. 3:**
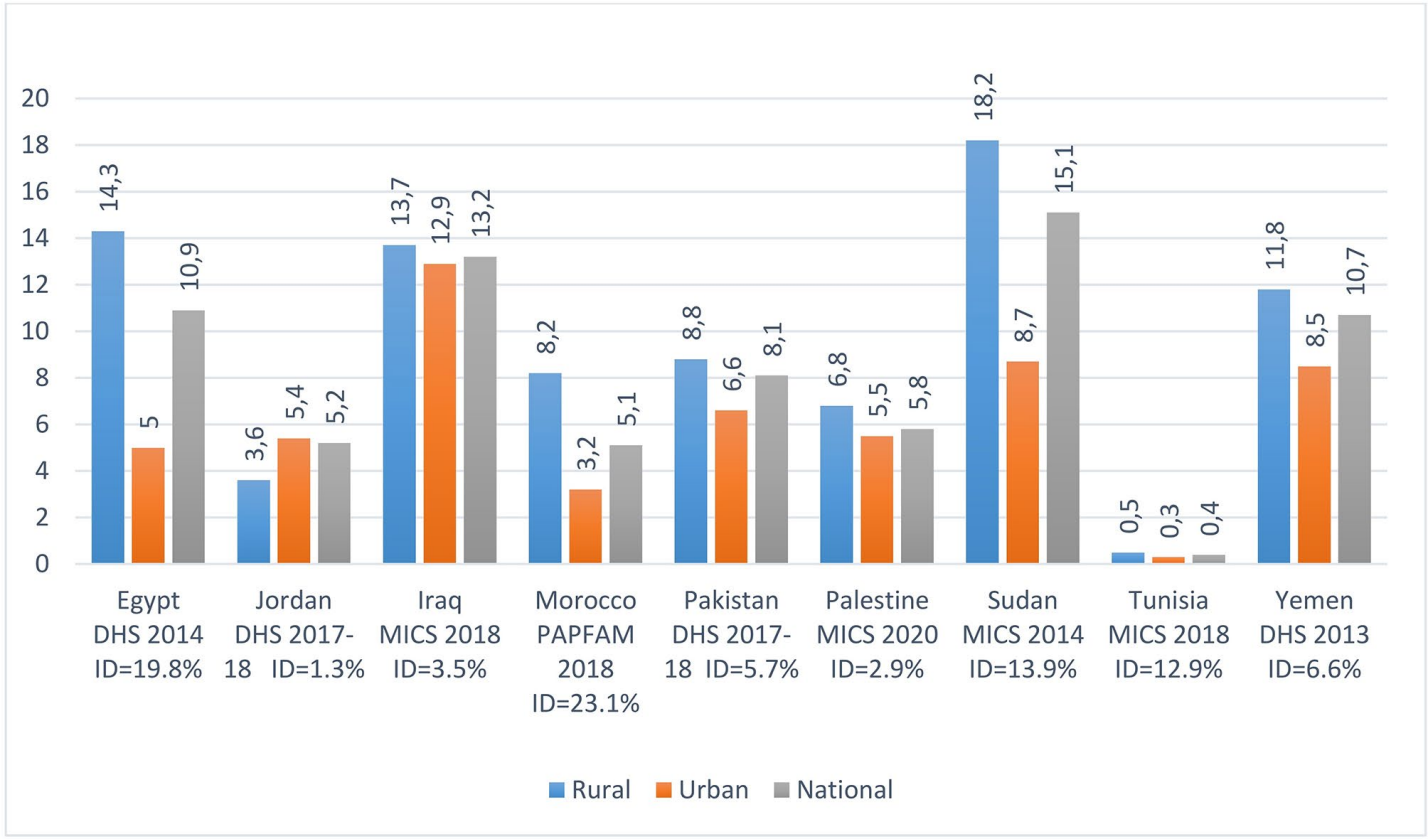
% of adolescent childbearing by milieu of residence (urban–rural) in nine countries

As indicated by Fig. 3 below, the prevalence of adolescent childbearing is higher in rural areas than in urban ones in eight countries out of nine. In Sudan, the percent of adolescent girls who have begun childbearing is more than twice higher in rural areas (18.2%) than in urban areas (8.7%). The ratio reaches nearly 3 in Morocco (8.2% in rural zones vs. 3.2% in urban zones) and in Egypt (14.3% in rural zones vs. 5% in urban zones). The gaps are confirmed by the high values of the Index of Dissimilarity (ID) which reach 12.9%, 13.9%, 19.8% and 23.1% in Tunisia, Sudan, Egypt and Morocco, respectively.

Jordan is the only country where the percentage of adolescent pregnancy is greater in urban (5.4%) than in rural areas (3.6%). No explanation was given by the DHS 2017–2018 report for this Jordanian peculiarity. In fact, the four demographic health surveys carried out in 2002, 2007, 2012 and 2017–2018 produced similar urban–rural figures for the percent of adolescent pregnancy (4.8% vs. 2.2%, 4.3% vs. 2.9%, 5.0% vs. 2.4% and 5.4% vs. 3.6%). One possible explanation can be taken from the UNFPA & Population Council report published in 2009 which indicates that the percent of 15–19 years old females who were currently married or in a union was much higher in urban zones (6.4%) than in rural zones (3.8%). Another explanation is given in terms of age at first marriage or union for 20–24 year-old females, showing that 3.4% adolescents were married at age 15 in urban areas compared to 1.0% in rural areas. Finally, a third proof that may explain why Jordan have more adolescent births in urban than in rural areas can be given by the fact that the percent of 15–24 years old ever-married females whose spouse ever forced sex is greater in urban than in rural areas [23].

### Adolescent childbearing by education level in 10 countries of the WHO-EMR

**Fig. 4:**
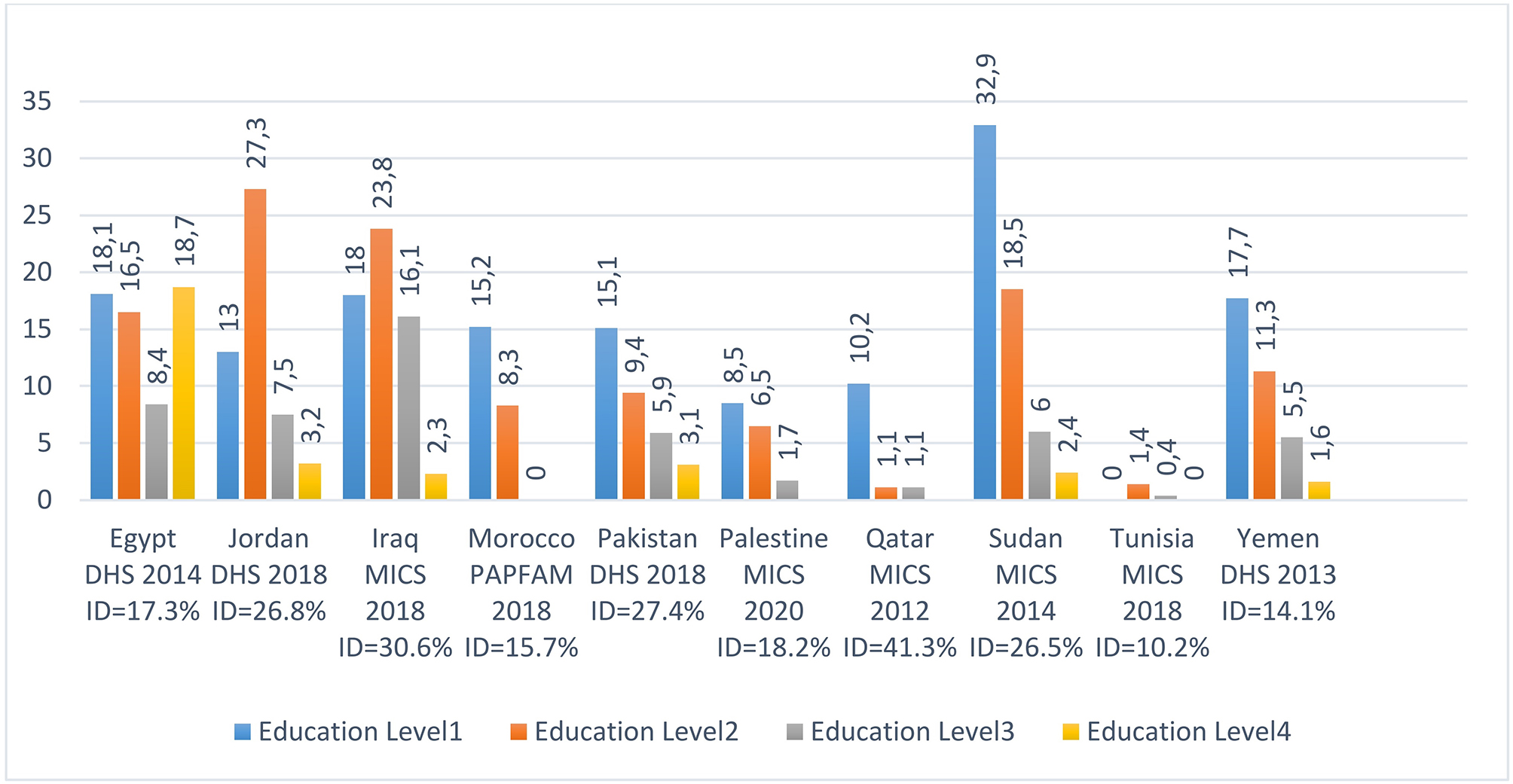
% of adolescent childbearing by education level in nine countries

The level of education affects crucially early marriage and teenage pregnancy/motherhood. Figure 4 shows that, in general, young women with no or low level of education are more exposed to pregnancy or motherhood than their counterparts with secondary/higher level. The biggest gaps are seen in Sudan where the percent of adolescent girls with no education (32.9%) is nearly 14 times higher than the percent of adolescent girls with higher level of education (2.4%) and in Morocco where it varies from 15.2% among no educated girls to zero among girls with secondary level or plus. The ten indices of dissimilarity are all greater than 10%, reaching 26.5%, 26.8%, 27.4%, 30.6% and 41.3% in Sudan, Jordan, Pakistan, Iraq and Qatar respectively. This inequity distribution shows clearly the effect of education on early marriage and the consequent pregnancy and motherhood among adolescent girls. The comparison between countries should be cautious given that levels of education are not defined by the same way in the ten countries considered.

Decision makers in WHO Eastern Mediterranean countries are called to act urgently to end child marriage by assuring the maximum years of education especially to disadvantaged girls living in rural and remote zones.

It should be stressed, however, that the general trend does not apply for the data from Egypt. Indeed, the highest percent of Egyptian adolescent childbearing (18.7%) is associated with secondary complete/higher education (level 4), exceeding even the percent of adolescent childbearing among the non-educated girls (level 1). We could not find in the literature any explanation for this singular situation, rare in the world. One possible explanation can be deduced from the Human Rights Watch Report, 2022 which indicates that Egyptian married students who are pregnant or are mothers are reportedly able to continue their schooling through home schooling while pregnant girls and young mothers face high levels of stigma from their peers at school, from school officials, and from their community worldwide [24].

### Adolescent childbearing by wealth quintiles in 9 countries of the WHO–EMR

**Fig. 5:**
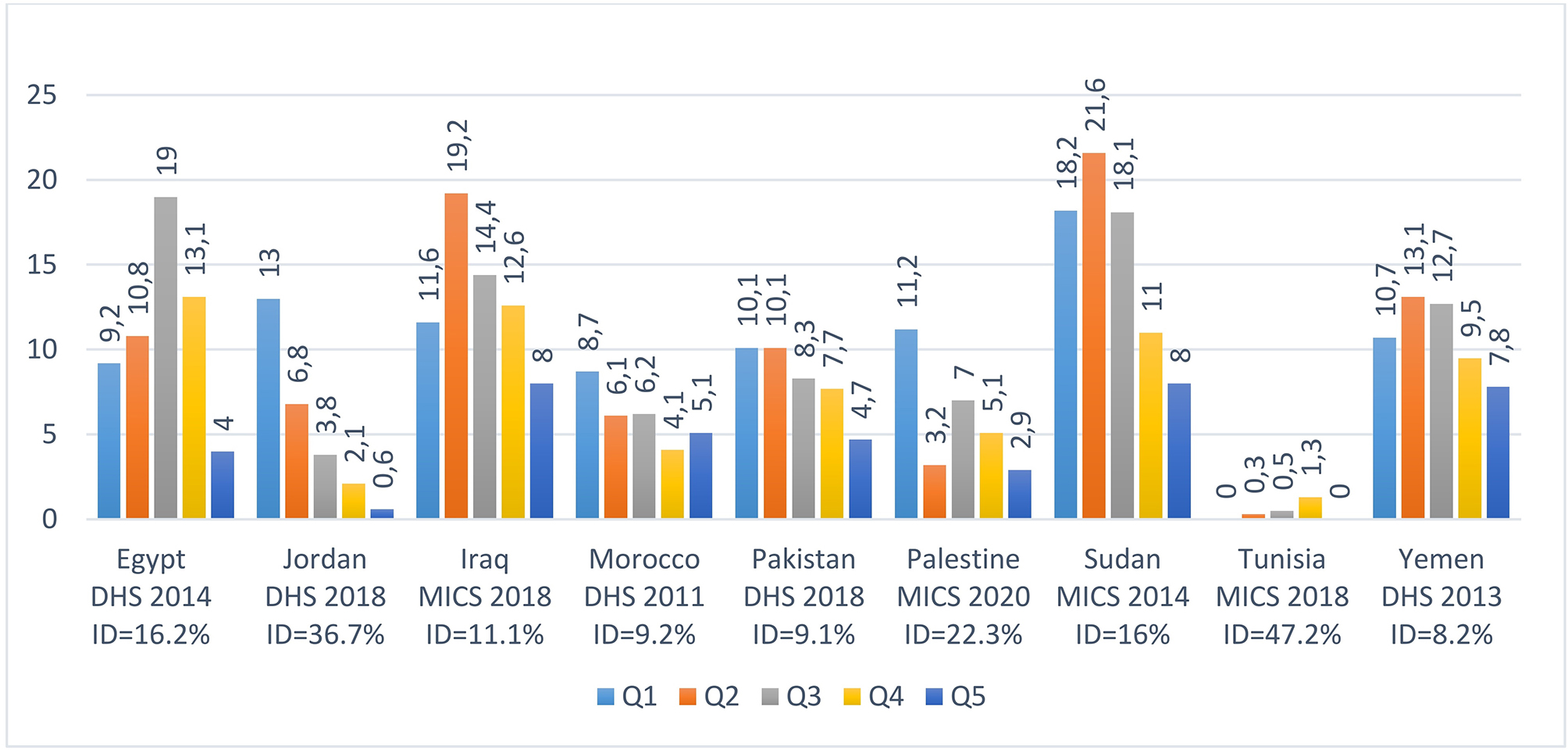
Inequity in the percentage of adolescent girls who have begun childbearing within and between nine countries of the WHO–Eastern Mediterranean Region by wealth quintiles **ID**: index of Dissimilarity, **Q1**: poorest quintile, **Q2**: second quintile, **Q3**: third quintile, **Q4**: fourth quintile, **Q5**: richest quintile.

Figure 5 shows that crucial differences are also induced by wealth quintiles. For example, the percentage of Jordanian adolescents who have begun childbearing increases from 0.6% in the richest quintile (Q5) to 13% in the poorest quintile (Q1) indicating a ratio of nearly 22. Large differences are also seen in Egypt between teenage pregnancy and motherhood in the richest quintile (4%) compared to the third quintile (19%). For the nine countries, gaps are confirmed by values of the index of dissimilarity which are between 8% and 10% in three countries (Morocco, Pakistan and Yemen), between 10% and 20% in three countries (Egypt, Iraq and Sudan) and greater than 20% in three countries (Jordan, Palestine and Tunisia).

### Adolescent childbearing by regions in some countries of the WHO–EMR

**Fig. 6:**
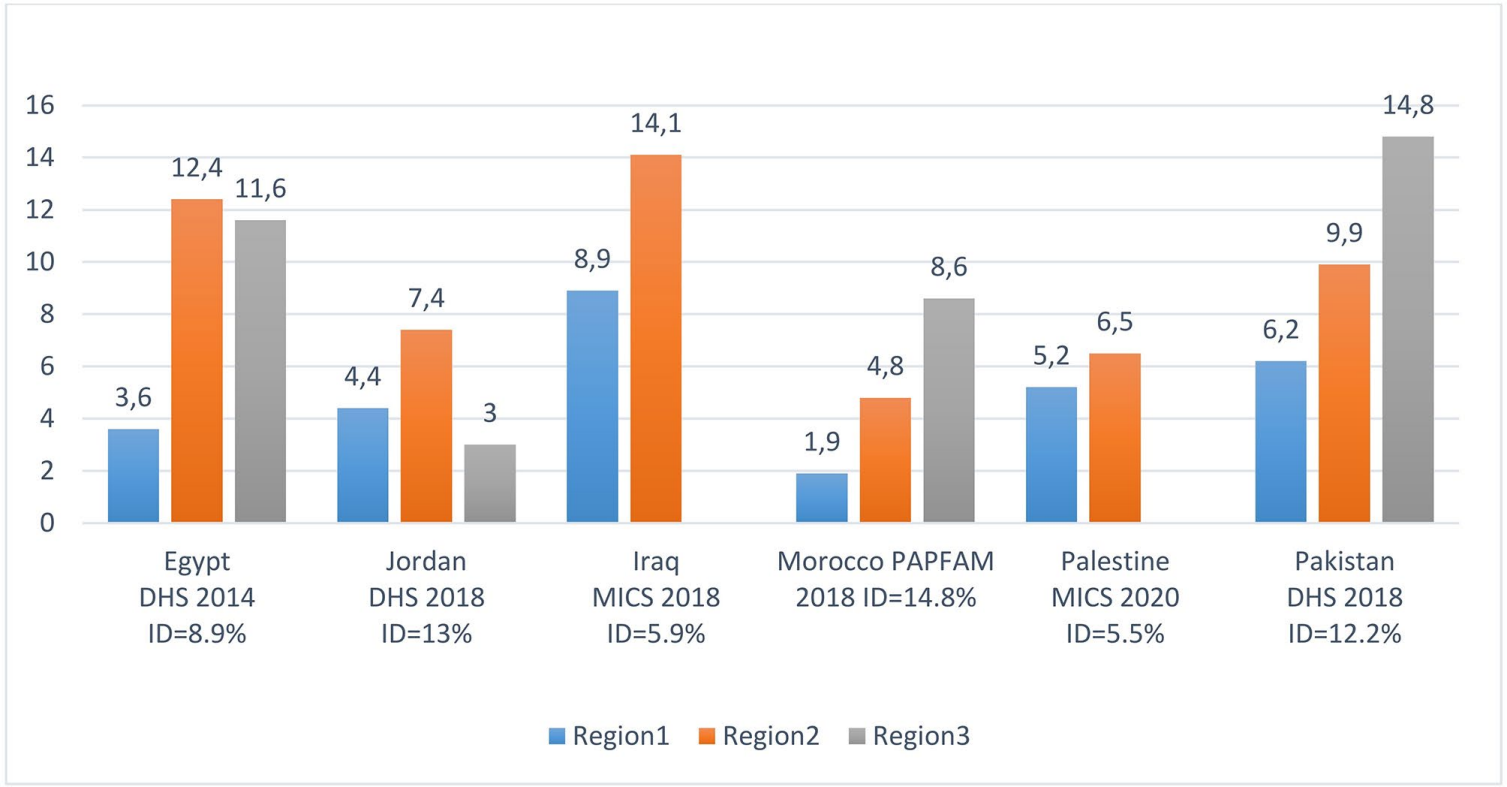
: Inequity in the percentage of adolescent girls who have begun childbearing in countries of the WHO-Eastern Mediterranean Region by country’s regions

As illustrated by Fig. 6, except in Palestine, teenage pregnancy/motherhood varies considerably by region in each country of the WHO Eastern Mediterranean Region. In Morocco, the percent of adolescent girls who have begun childbearing is more than 4 times higher in the region of BéniMellal–Khénifra (8.6%) than in the “Oriental” region (1.9%). Similarly, the ratio reaches 3.4 between the Lower-Egypt region (12.4%) and the urban governorates (3.6%). This important information should be exploited by the governments to end or at least to limit child marriage by tackling the problem spatially in the regions while targeting first areas where the percentage of early marriage is higher.

### Adolescent childbearing by nationality in some countries of WHO–EMR

**Fig. 7:**
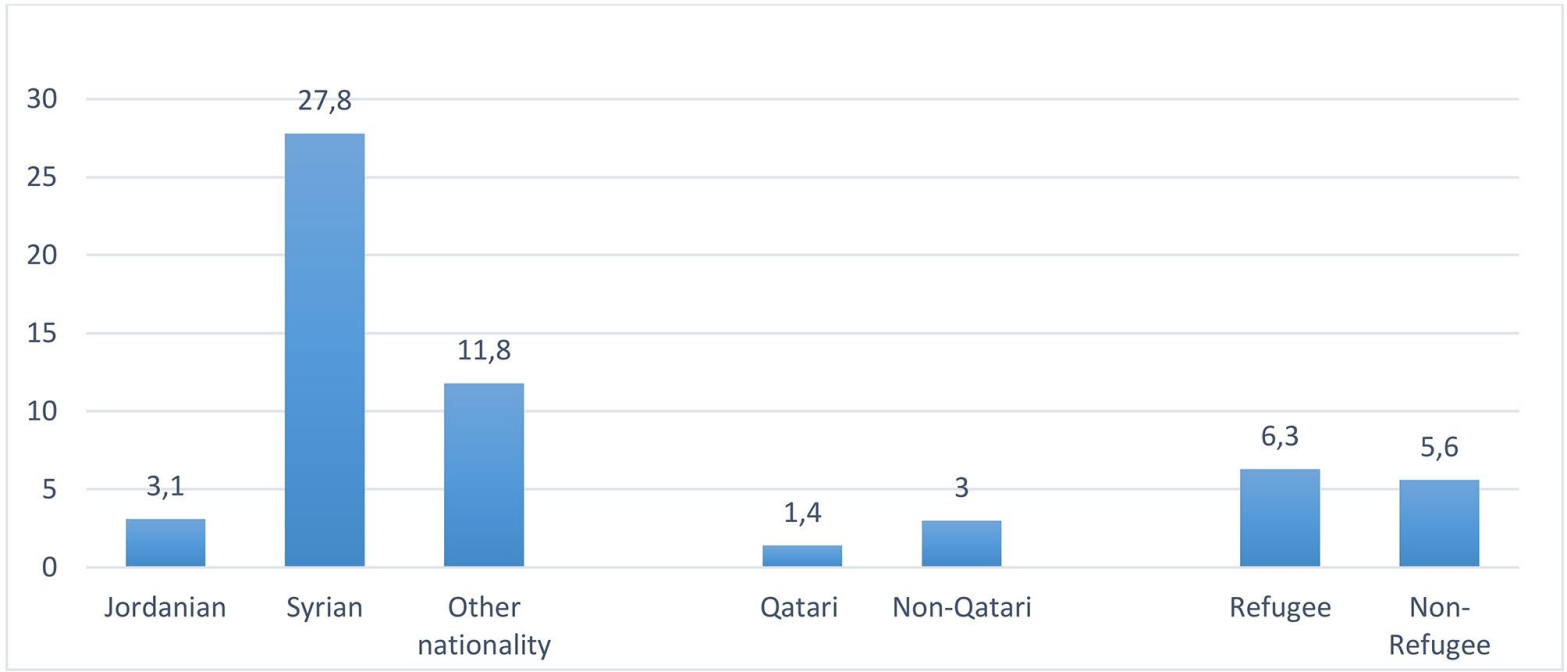
Inequity in the percentage of adolescent girls who have begun childbearing in countries of the WHO–Eastern Mediterranean Region by nationality or Refugee status Jordan DHS 2018: ID = 37.7%, Qatar MICS 2012: ID = 18.3% and Palestine MICS 2010: ID = 3%.

Finally, Gulf countries are known to exhibit large differences between nationals and non-nationals in different socio-economic domains. Concerning the problem of adolescent childbearing, scarce available data show that non-Qatari girls (3%) are more than twice exposed to teenage pregnancy/motherhood than Qatari girls (1.4%) while in Jordan, Syrian girls (27.8%) are nearly 9 times more likely to be pregnant or mothers compared to Jordanian adolescent girls (3.1%). In Palestine, a slight difference is seen between refugee girls (6.3%) and non-refugee girls (5.6%) (Fig. 7).

## Discussion

Early marriage causes a multitude of harm to girls, their offspring and the whole society. As stressed by the Executive Director of UNFPA in its foreword to the report entitled: Girlhood Not Motherhood, *“When a girl becomes pregnant, her present and future change radically, and rarely for the better. Her health is endangered, her education and job prospects abruptly end and her vulnerability to poverty and exclusion multiplies. Pregnancy before a girl is physically, developmentally and socially ready jeopardizes her right to a safe, successful transition into adulthood”* [25].

Although adolescent childbearing is a global problem occurring in high-, middle-, and low-income countries, this crucial phenomenon is more likely to affect disadvantaged girls living in marginalized communities and suffering from poverty, illiteracy and lack of employment opportunities [1].

**Fig. 8:**
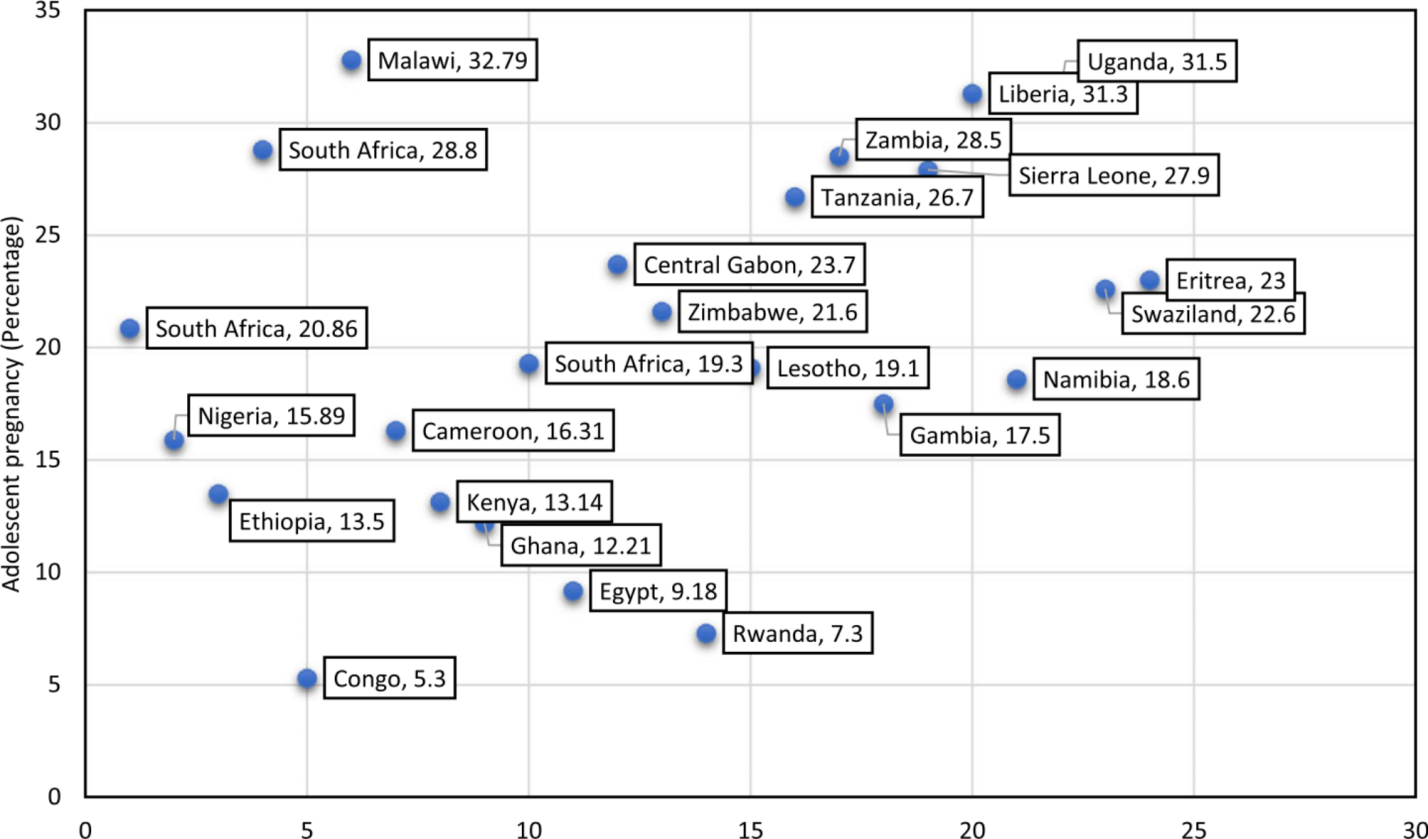
Distribution of pooled prevalence of adolescent pregnancy in 24 African countries, 2003 to 2018

The results given in the previous section show that the prevalence of adolescent childbearing in the ten WHO Eastern Mediterranean countries is 8.8%, with a large variation between and within these countries. Prevalence of childbearing in WHO Eastern Mediterranean countries is, however, lower than in African countries and in the Latin America and the Caribbean (LAC) region. Indeed, in 2018, Kassa et al. published a systematic review and meta-analysis on prevalence and determinants of adolescent pregnancy in Africa. Analysing data provided by 52 studies from 24 African countries yielded an average prevalence of adolescent pregnancy in Africa of 18.8% (95% CI: 16.7–20.9%). Although the comparison between countries should be cautious given that studies were carried out over a period of 15 years (2003 to 2018), the prevalence of adolescent pregnancy varied from 5.3% in Congo to 32.79% in Malawi (Fig. 8) [26]. The review also found that the main factors associated with adolescent pregnancy were lack of parent to adolescent communication on sexual and reproductive health issues (OR: 2.88), not attending school (OR: 2.49), rural residence (OR: 2.04), no maternal education (OR: 1.88) and no father’s education (OR: 1.65) [26].

Reproduced with kind permission from the first author of the paper.

According to a technical consultation on the problem of adolescent pregnancy held in 2017 by PAHO, UNFPA and UNICEF, it was estimated that 15% of all pregnancies in Latin America and the Caribbean region occur amongst girls younger than 20 years of age with large inequities by education level, wealth quintiles and ethnicity within countries [5].

In the ten WHO Eastern Mediterranean countries considered in this study, the percentage of young women who have begun childbearing indicates huge gaps within each country according to social determinants of health like milieu of residence (urban-rural), level of education, wealth quintiles, country’s region and nationality.

These results suggest that pragmatically, for an efficient strategy to reduce early marriage and adolescent pregnancy, decision makers should act on social determinants like milieu of residence (urban–rural), wealth quintiles, education level, territoriality (regions) and nationality. This suggestion is supported by the fact that adoption of theoretical conventions and laws are insufficient. Indeed, according to the UNICEF report on child marriage in the Middle East and North Africa, ‘‘*there is often a contradiction between national law and customary and religious law under which many marriages are conducted. Many national constitutions provide exceptions for personal or family law, facilitating many other avenues for child marriages to be practised’’* [27]. For example, the minimum legal age of marriage is 18 in Jordan and Morocco but judges in these two countries may “exceptionally” allow girls and boys to marry at an earlier age. In Morocco, the family code (Moudawana) which was adopted in 2004, fixed the minimal age of marriage at 18 years for both girls and boys [28]. About 500,000 early formal marriages were, however, registered since the launch of this family code 17 years ago. Moreover, there are also informal marriages known as ‘‘Orfi ‘’ marriages or with ‘‘Al fatiha’’ or even by ‘‘contracts’’ often concluded between men living abroad and poor fathers of children, in return for a sum of money [29].

As indicated by the report of the Commission on Social Determinants of Health in the Eastern Mediterranean Region, this region “*has the highest levels of gender inequality measured by the Gender Inequality Index compared with other global regions*” [15]. Consequently, countries of this region are urged to ratify the Convention on the Elimination of all Forms of Discrimination against Women (CEDAW), knowing that child marriage is one of the silent exacerbated forms of discrimination against young women [27].

The inequity phenomenon illustrated in this paper in terms of adolescent childbearing gaps according to different social determinants of health in countries of the WHO-EMR, constitutes a tiny component of a more structural problem of socio-economic inequalities, territorial disparities and health inequities in the quasi-totality of the WHO–EMR countries [30–38].

## Conclusion

Analysis of available disaggregated data by residence (urban–rural), wealth quintiles, education level, territoriality (regions) and nationality shows clearly that decision makers in WHO–EMR countries should urgently and efficiently act on social determinants of health in the short and medium term to limit pregnancy before the age of 20 years, reduce unsafe abortion among adolescents, increase the use of contraception by adolescents at the risk of unintended pregnancy and support adolescents during pregnancy, labour and postpartum [1, 12, 13, 39]. Meanwhile, a long-term action should aim to end the unacceptable phenomenon of early marriage and teenage childbearing or at least to limit it as far as possible [31, 32, 40]. Research studies have proved that disadvantaged girls coming mainly from marginalised groups and poor families living in remote rural zones are the most exposed to early marriage and adolescent childbearing. Moreover, they have a low (or no) level of education and are unlikely to be reached by interventions [5, 12, 13, 26, 40]. Consequently, they must be targeted seriously by urgent and efficient strategies.

## Limitations

In this study we could not include the following data: (1) Afghanistan DHS (2015) because data were removed [41], (2) Oman MICS 2014 due to unavailability of disaggregated data [42], (3) Qatar MICS 2022 and Lebanon MICS 2022 for which complete data have not yet been published.

It should also be stressed that all the secondary data sources (DHS, MICS, PAPFAM) we used concentrate on childbearing in adolescents aged 15–19 years and consequently, the actual rate of adolescent pregnancy should be higher due the missing numbers corresponding to marriages/births of girls aged 14 years or younger.

## Data Availability

Data used are accessible through DHS and MICS.

## Declarations

### Author information

The author is an honorary professor at the University Mohamed Premier, Oujda, Morocco. He has spent about 20 years doing research in mathematical modelling, health equity and SDH. He is also a Visiting Professor at Emirates Aviation University.

## References

[1] World Health Organisation. Adolescent pregnancy. https://www.who.int/news-room/fact-sheets/detail/adolescent-pregnancy. Accessed 2 Jan 2022

[2] World atlas. World Facts: Highest Teen Pregnancy Rates Worldwide 2015 [updated April 25, 2017]. https://www.worldatlas.com/articles/highest-teen-pregnancy-rates-worldwide.html. Accessed 20 Feb 2023.

[3] The World Bank. Adolescent fertility rate (births per 1,000 women ages 15–19) 2019. https://data.worldbank.org/indicator/SP.ADO.TFRT. Accessed 20 Feb 2023.

[4] Yakubu I, Salisu WJ (2018). Determinants of adolescent pregnancy in sub-saharan Africa: a systematic review. Reprod Health.

[5] Caffe S, Plesons M, Camacho AV (2017). Looking back and moving forward: can we accelerate progress on adolescent pregnancy in the Americas?. Reprod Health.

[6] USAID. The Demographic and Health Survey program, Rockville. USAID; 2021. Egypt DHS 2014. https://dhsprogram.com/pubs/pdf/FR302/FR302.pdf. Accessed 20 Dec 2021.

[7] USAID. The Demographic and Health Survey program, Rockville USAID; 2021. Jordan DHS 2017-18. https://dhsprogram.com/pubs/pdf/FR346/FR346.pdf. Accessed 15 Dec 2021.

[8] USAID. The Demographic and Health Survey program, Rockville. USAID; 2021. Pakistan DHS 2017. https://dhsprogram.com/pubs/pdf/FR354/FR354.pdf. Accessed 10 Dec 2021.

[9] USAID. The Demographic and Health Survey program, Rockville. USAID; 2021.Yemen DHS 2013. https://dhsprogram.com/pubs/pdf/FR296/FR296.pdf. Accessed 25 Dec 2021.

[10] Kingdom of Morocco, Ministry of Health, Rabat. PAPFAM 2018. https://www.sante.gov.ma/Documents/2020/03/Rapport%20ENPSF%202018%202ième%20édition.pdf. Accessed 2 Dec 2021.

[11] UNICEF, Early, Childbearing. Early childbearing can have severe consequences for adolescent girls. https://data.unicef.org/topic/child-health/adolescent-health/. Accessed 20 Feb 2023.

[12] UNFPA. (2020). Advancing the Evidence Base Strategies to End Child Marriage and Support Married Girls Meeting Report. https://www.unfpa.org/sites/default/files/resource-pdf/GP_Child-marriage-global-research-meeting_August2020.pdf. Accessed 9 Dec 2021.

[13] UNFPA-UNICEF Global Programme to End Child Marriage. Driving action to reach the girls at greatest risk. https://www.unicef.org/protection/unfpa-unicef-global-programme-end-child-marriage. Accessed 2 Jan 2022.

[14] CSDH. (2008). Closing the gap in a generation: health equity through action on the social determinants of health. Final Report of the Commission on Social Determinants of Health. Geneva, World Health Organisation. https://apps.who.int/iris/bitstream/handle/10665/43943/9789241563703_eng.pdf. Accessed 12 Dec 2021.

[15] CSDH-EMR 2021. Build back fairer: achieving health equity in the Eastern Mediterranean Region.Report of the Commission on the Social Determinants of Health in the Eastern Mediterranean Region. 10.1016/S0140-6736(21)00710-8 33798501

[16] World Health Organisation. WHO guidelines on Preventing early pregnancy and poor reproductive outcomes among adolescents in developing countries. https://www.who.int/publications/i/item/9789241502214. Accessed 21 Feb 2023.

[17] Kingdom of Morocco, Ministry of Health, Rabat. PAPFAM 2011. https://www.sante.gov.ma/Documents/Enquête%20.pdf. Accessed 30 Dec 2021.

[18] UNICEF. Multiple Indicator Cluster Surveys (MICS). UNICEF 2021. Iraq MICS 2018. https://mics.unicef.org/surveys. Accessed 4 Oct 2021.

[19] UNICEF 2021. UNICEF. Multiple Indicator Cluster Surveys, Palestine MICS. 2021. https://mics-surveys-prod.s3.amazonaws.com/MICS6/Middle%20East%20and%20North%20Africa/State%20of%20Palestine/2019-2020/Survey%20findings/State%20of%20Palestine%202019-20%20Survey%20Findings%20Report_v2_English.pdf. Accessed 9 Dec 2021.

[20] UNICEF 2022. UNICEF. Multiple Indicator Cluster Surveys (MICS), Qatar MICS. 2012. https://mics-surveys-prod.s3.amazonaws.com/MICS4/Middle%20East%20and%20North%20Africa/Qatar/2012/Final/Qatar%202012%20MICS_English.pdf. Accessed 10 Oct 2021.

[21] UNICEF 2021. UNICEF. Multiple Indicator Cluster Surveys (MICS), Sudan MICS. 2014. https://mics-surveys-prod.s3.amazonaws.com/MICS5/Middle%20East%20and%20North%20Africa/Sudan/2014/Final/Sudan%202014%20MICS_English.pdf. Accessed 10 Oct 2021.

[22] UNICEF. Multiple Indicator Cluster Surveys (MICS). UNICEF 2021, Tunisia MICS. 2018. https://mics-surveys-prod.s3.amazonaws.com/MICS6/Middle%20East%20and%20North%20Africa/Tunisia/2018/Survey%20findings/MICS%20Tunisia%202018-SFR_French.pdf. Accessed 20 Dec 2021.

[23] UNFPA & Population Council. The adolescent experience in-depth: using data to identify and reach the most vulnerable young people Jordan 2007. https://www.popcouncil.org/uploads/pdfs/PGY_AdolDataGuides/Jordan2007.pdf. Accessed 21 Feb 2023.

[24] Human Rights Watch. Across Africa, many Young Mothers Face Education Barriers., 2022. https://www.hrw.org/news/2022/08/30/across-africa-many-young-mothers-face-education-barriers. Accessed 20 Feb 20 2023.

[25] UNFPA. Girlhood, not motherhood: Preventing adolescent pregnancy. New York, UNFPA., 2015. https://www.unfpa.org/sites/default/files/pub-pdf/Girlhood_not_motherhood_final_web.pdf. Accessed 23 Dec 2021.

[26] Kassa GM, Arowojolu AO, Odukogbe AA (2018). Prevalence and determinants of adolescent pregnancy in Africa: a systematic review and Meta-analysis. Reproductive Health.

[27] UNICEF. (2017). Child marriage in the Middle East and North Africa. https://www.unicef.org/mena/media/1786/file/MENA-ChildMarriageInMENA-Report.pdf.pdf Accessed 20 Oct 2021.

[28] Kingdom of Morocco, Ministry of Justice. Family Code ‘‘Moudawana’’. https://adala.justice.gov.ma/production/legislation/fr/civil/code%20de%20la%20famille%20maroc%20texte.htm. Accessed 30 Oct 2021.

[29] Boutayeb A (2022). Evolution of early marriage and adolescent childbearing by social determinants of health in Morocco. Ann Public Health Preservative Med.

[30] Ben Romdhane H, Grenier FR (2009). Social determinants of health in Tunisia: the case-analysis of Ariana. Int J Equity Health.

[31] Boutayeb A (2006). Social inequalities and health inequity in Morocco. Int J Equity Health.

[32] Boutayeb A. Social Determinants of Health and Health Equity in the WHO African Region. In: Boutayeb A, editor. Disease Prevention and Health Promotion. Springer Nature Switzerland AG; 2020. p.11–28. doi: 10.1007/978-3-030-34702-4-2

[33] Boutayeb A. Inequalities within and between the WHO Eastern Mediterranean countries. In: Boutayeb A and Maamri A, editors. Health Inequity: A Crucial Issue Worldwide. Cambridge Scholars Publishing, Newcastle upon Tyne, NE6 2PA, UK; 2023. p. 1–39. ISBN: 978-1-5275-9371-8, https://www.cambridgescholars.com/product/978-1-52759371-8

[34] Boutayeb A, Lamlili M, Boutayeb W (2018). Health inequity and territorial disparity in under-five mortality between and within WHO-EMR countries. Int J Manag Appl Sci.

[35] Boutayeb A, Boutayeb S, Boutayeb W (2013). Multi-morbidity of non communicable diseases and equity in WHO Eastern Mediterranean Countries. Int J Equity Health.

[36] Khadr Z, Rashad H, Shawky S. Health Inequalities in Jordan and their social determinants: evidence and policy implications. The Social Research Center of the American University in Cairo and the World Health Organization/ Jordan; 2019.

[37] Kotti AB, Cherif A, Elloumi A (2021). The social roots of Health Inequity in Tunisia: a preliminary study on the Social Determinants of Health Inequity. Adv Soc Sci Res J.

[38] Rashad H, Shawky S, Khadr Z. Reproductive Health Equity in the Arab Region: Fairness and Social Success, Regional Report 2019. The Social Research Center, The American University in Cairo, UNFPA/ASRO. https://documents.aucegypt.edu/Docs/src/Reproductive-Health-%20Equity-in-the-Arab-Region.pdf

[39] UNESCO. (2021). International technical guidance on sexuality guidance. An evidence informed approach. https://www.unfpa.org/sites/default/files/pub-pdf/ITGSE.pdf. Accessed 29 Dec 2021.

[40] Kirchengast S, Teenage Pregnancies. A Worldwide Social and Medical Problem, An Analysis of Contemporary Social Welfare Issues. Rosario Laratta Intech Open. 2016; doi: 10.5772/65462. https://www.intechopen.com/chapters/52475. Accessed 2 Dec 2021.

[41] USAID. The Demographic and Health Survey program, Rockville USAID; 2021. Afghanistan DHS 2015. https://dhsprogram.com/Countries/Country-Main.cfm?ctry_id=71&c=Afghanistan&Country=Afghanistan&cn=&r=4. Accessed 20 Dec 2021.

[42] UNICEF 2021. UNICEF. Multiple Indicator Cluster Surveys (MICS), Oman MICS. 2014. https://mics-surveys-prod.s3.amazonaws.com/MICS5/Middle%20East%20and%20North%20Africa/Oman/2014/Key%20findings/Oman%202014%20MICS%20KFR_English.pdf. Accessed 27 Nov 2021.

